# Two Odorant-Binding Proteins of the Dark Black Chafer (*Holotrichia parallela*) Display Preferential Binding to Biologically Active Host Plant Volatiles

**DOI:** 10.3389/fphys.2018.00769

**Published:** 2018-07-18

**Authors:** Qian Ju, Xiao Li, Xiao-Qiang Guo, Long Du, Chen-Ren Shi, Ming-Jing Qu

**Affiliations:** Shandong Peanut Research Institute, Qingdao, China

**Keywords:** *Holotrichia parallela*, odorant-binding proteins, host plant volatiles, reverse chemical ecology, (Z)-3-hexenyl acetate

## Abstract

The dark black chafer (DBC), *Holotrichia parallela*, is an important pest of multiple crops. Insect host-searching behaviors are regulated by host plant volatiles. Therefore, a better understanding of the mechanism linking the chemosensory system to plant volatiles at the molecular level will benefit DBC control strategies. Based on antenna transcriptome data, two highly expressed antenna-specific odorant-binding proteins (HparOBP20 and 49) were selected to identify novel DBC attractants using reverse chemical ecology methods. We expressed these proteins, mapped their binding specificity, and tested the activity of the plant volatiles in the field. The ligands used in the binding specificity assays included 31 host-plant-associated volatiles and two sex pheromone components. The results showed that (1) HparOBP20 and 49 are involved in odor recognition; (2) these proteins bind attractive plant volatiles strongly and can therefore be employed to develop environmentally friendly DBC management strategies; and (3) the green-leaf volatile (Z)-3-hexenyl acetate shows a high binding affinity to HparOBP20 (Ki = 18.51 μM) and HparOBP49 (Ki = 39.65 μM) and is highly attractive to DBC adults, especially females. In the field test, a (Z)-3-hexenyl acetate trap caught an average of 13 ± 1.202 females per day, which was significantly greater than the corresponding male catch (*F*2,6 = 74.18, *P* < 0.0001). (Z)-3-Hexenyl acetate may represent a useful supplement to the known sex pheromone for DBC attraction. In the present study, the binding characteristics of two HparOBPs with host plant volatiles were screened, providing behaviourally active compounds that might be useful for DBC control, based on reverse chemical ecology.

## Introduction

The dark black chafer (DBC), *Holotrichia parallela* Motschulsky (Coleoptera: Scarabaeidae), is an important pest in agriculture and forestry. DBC larvae, often referred to as grubs, live in soil and can cause significant damage to peanut, sweet potato, soybean, corn, and various other vegetable crops as well as to turf and ornamental species (Ju et al., 2012; Shan et al., 2014). Due to its cryptic and subterranean nature, this beetle is difficult to control. The main tactic employed for DBC management is chemical control, which has environmentally detrimental consequences, such as residual toxicity, environmental contamination, and insecticide resistance. Mass trapping using sex pheromone-based attractants is an environmentally friendly control tactic and has become well established. However, this tactic has several shortcomings, including a male response bias to the sex pheromone traps and a short duration of residual activity (Reddy and Guerrero, 2004; Said et al., 2005). Similar to insect pheromones, plant volatiles are important signaling compounds that regulate insect behavior and exhibit potential as natural pesticides, lures, or antifeedants (Hanks et al., 2012; Hanks and Millar, 2013; Jung et al., 2013; Collignon et al., 2016; Wang F. et al., 2016; Wang Y.L. et al., 2016). Therefore, studies addressing the physiological and molecular basis of host plant selection could serve as an important basis for developing novel control tactics for the DBC (Koczor et al., 2012).

The interaction between plant volatiles and the insect olfactory system plays a critical role in the initial step of insect host orientation (Liu et al., 2015; Brito et al., 2016; Sun et al., 2017a). Plant volatiles consist of various classes of chemicals, such as green-leaf volatiles, general odorants and terpenoids (Aartsma et al., 2017). Due to the great diversity of plant volatiles, behavioral response methods for selecting active host plant volatiles require a great deal of time and effort. In this context, the reverse chemical ecology approach is gaining importance (Mao et al., 2010; Jayanthi et al., 2014), as it narrows down the number of odorant candidate compounds based on their binding affinity to olfactory proteins, saving time and reducing research costs compared with conventional trial-and-error screening performed in the field (Leal, 2017). Odorant-binding proteins (OBPs) are one of the major types of peripheral olfactory proteins involved in the reception of odorants in insects (Vogt et al., 1985; Klein, 1987; Leal, 2013). The physiological functions of insect OBPs have been described based on biochemical, biophysical, structural biology and kinetic studies (Sandler et al., 2000; Horst et al., 2001; Leal et al., 2005; Zhu et al., 2017), and it is clear that OBPs are important for transporting odorants through the sensillar lymph and increase the sensitivity of the olfactory system (Pelosi et al., 2014; Leal, 2017). The role of OBPs in the transport of molecules in insect antennae was described for the first time in Lepidoptera using male *Antheraea polyphemus* antennae (Vogt and Riddiford, 1981). Knockdown studies have demonstrated that DmelOBP76a (LUSH) is necessary for the olfactory process in *Drosophila melanogaster* (Xu et al., 2005; Laughlin et al., 2008). Furthermore, behavioural assays in *Drosophila* mutants (Matsuo et al., 2007; Swarup et al., 2011) and aphids (Qiao et al., 2009; Sun et al., 2012) have indicated that OBPs are involved in semiochemical detection. Previous studies have shown that a blend of volatiles derived from host plants can bind to OBPs and be used as a luring agent. A good example is provided by *Loxostege sticticalis* OBP2, which has been shown to exhibit a high affinity to host plant volatiles (Yin et al., 2012). OBP1 of *Grapholita molesta* exhibits dual functions in the recognition of host plant volatiles (Li et al., 2016). Two *Spodoptera exigua* OBPs share a common odorant-response spectrum, with a considerable binding affinity to host odorants (Liu et al., 2017). Binding assays of two OBPs from *H. oblita* with various compounds showed that benzoates (leaf volatiles from host plants) fit inside the OBPs (Deng et al., 2012).

However, little is known about the molecular mechanisms underlying the interactions between DBCs and the odorous environment of their host plants. To date, only one report has described the binding functions of two OBPs in the DBC (Ju et al., 2012). Using a rapid amplification of cDNA ends (RACE) approach, the HparOBP1 and HparOBP2 genes were identified, and their ligand-binding properties were examined. Due to recent transcriptome projects, a large number of insect OBP sequences are available. Additionally, 25 OBP genes were obtained from the DBC whole-body transcriptome (Ju et al., 2014). However, the OBPs predicted from insect whole-body genomes are all unlikely to represent true olfactory proteins. In *D. melanogaster*, for instance, the OBP gene family comprises as many as 51 putative OBPs, but only seven of them have been demonstrated to be expressed specifically in adult olfactory organs (Galindo and Smith, 2001). At present, investigation of the antennal transcriptome is an effective way to find functional OBPs binding to plant volatiles. In this study, we identified the OBP genes expressed in DBC antennae using the transcriptome sequencing approach. Two HparOBPs were selected based on their specific phylogenetic position and antenna-specific expression pattern to determine their ligand-binding properties. Furthermore, the attractive properties of ligands binding to the two HparOBPs were verified in behavioral responses tests and field evaluations. Taken together, our results extend the knowledge of OBP genes in the DBC and pave the way for the development of novel environmentally friendly control tactics for DBC management.

## Materials and Methods

### Insects and Insect Maintenance

Adults DBCs were collected from the field of the experimental station at Shandong Peanut Research Institute, Qingdao, China. The beetles were separated into males and females and were reared with fresh elm tree (*Ulmus pumila* L.) leaves in a rotating chamber with aerating meshes. The relative humidity in the rearing chamber was maintained at 18–20%. Fresh antennae were obtained from both males and females for experimentation.

### Transcriptome Sequencing

Total RNA was isolated from adult antennae (∼200 antennae from both males and females). The RNA was quantified using a NanoDrop spectrophotometer (Thermo, Franklin, TN, United States). The mRNA was subsequently used for cDNA synthesis as described by Rice et al. (2000). cDNA synthesis, library construction and sequencing, gene annotation and prediction, and OBP identification and confirmation were conducted as described in previous articles (Ju et al., 2014). Briefly, the double-stranded cDNAs were fragmented into segments of 300–500 bp via sonication, and the sonicated mixture was purified using Agencourt-AMPure beads (Beckman, Schaumburg, IL, United States). A cDNA library was then generated using the TruSeq^TM^ RNA Sample Prep Kit (Illumina, San Diego, CA, United States). The cDNA library was subsequently sequenced on the Illumina HiSeq 4000 sequencing platform. Raw read quality was assessed using FastQC^1^ prior to assembly, and Trimmomatic was used to filter adaptor sequences and trim reads bases with a PHRED quality score below 20. After adaptor filtering, the resulting reads were *de novo* assembled into contigs using the Trinity program. The ‘align and estimate abundance’ script in the Trinity package was used to align the reads and perform transcript abundance estimation using the RSEM method. The assembled contigs were further clustered using the TGI Clustering Tool (Pertea et al., 2003).

### RNA Isolation, CDNA Synthesis, and PCR Cloning

The cloning primers were designed using Primer Express 3.0 and are listed in Supplementary Table S1. PCR was carried out in a total volume of 50 μl containing 200 ng of cDNA template, 5 μl of 10× buffer, 4 mmol/L MgCl_2_, 0.8 μmol/L of each forward and reverse primer, 1 mmol/L dNTPs, and 2.5 U of Taq polymerase. The PCR program started at 95°C for 5 min for denaturation, followed by 25 cycles of 30 s at 95°C, 30 s at 60°C, and 30 s at 72°C, with a final extension at 72°C for 5 min.

### Phylogenetic Analysis

Both the novel OBP genes identified in this study and the reported OBP gene sequences retrieved from previous studies were included in the phylogenetic analysis (Ju et al., 2014; Li X. et al., 2015). Multiple alignments of OBP genes were generated using MAFFT alignment software version 7.215 (Katoh et al., 2009). Based on the capability for parallelizing computation, the IQ-TREE program version 1.5 was employed to construct a phylogenetic tree using the protein sequences of these OBP genes according to the maximum likelihood principle (Lam-Tung et al., 2015). The best protein substitution model was selected by the built-in model-selection function of the IQ-TREE program, and bootstrap support values from 1000 replicates were assessed with ultrafast bootstrap approximation.

### Fluorescence Competitive Binding Assay

Recombinant protein expression and purification were performed according to our previously reported protocols (Ju et al., 2012). Briefly, plasmid constructs containing the HparOBP genes were generated and transformed into Rosetta (DE3) competent cells for recombinant protein expression, and the resulting proteins were highly induced with 1 mM isopropyl ß-D-1-thiogalactopyranoside (IPTG) for 3–6 h at 37°C. Purification was performed via Ni ion affinity chromatography (GE Healthcare, Beijing, China), and the His-tag was removed using enterokinase for HparOBP20 or tobacco etch virus (TEV) protease for HparOBP49. Renaturation and extensive dialysis were performed as previously reported (Ju et al., 2012), and the size and purity of the recombinant proteins were verified through SDS-PAGE.

For the ligand-binding assays, 33 compounds (>95% purity, Sigma-Aldrich, Shanghai, China) were selected based on their previously reported isolation from DBC host plants (Cheng et al., 2010; Lee et al., 2010; Shi et al., 2011; Ju et al., 2012; Tang et al., 2012; Iqbal et al., 2014). We used an F-380 fluorescence spectrophotometer (Tianjin, China) to determine the results of the perform the binding assay at room temperature (25°C). The excitation wavelength was 337 nm, and the emission spectrum was recorded between 390 and 460 nm. *N*-phenyl-1-naphthylamine (1-NPN) is an effective fluorescent probe for insect OBP binding studies. First, we measured the constant emission of HparOBPs with 1-NPN, and titrated 2 μM proteins in 50 mM Tris-HCl (pH 7.4) with 1 mM 1-NPN in methanol to final concentrations ranging from 1 to 24 μM. Then, the affinities of other ligands were tested in competitive binding assays using 1-NPN as a fluorescent reporter at a concentration of 2 μM, while the concentration of each competitor ranged from 2 to 30 μM. We evaluated each bound chemical based on its fluorescence intensity, with the assumption that the protein was 100% active of 1:1 (protein/ligand) saturation. The binding curves were linearized using a Scatchard plot, and the dissociation constants of the competitors were calculated from the Scatchard plot of the binding data and the corresponding IC50 values based on the following equation: Ki = [IC_50_]/(1+[1-NPN]/K_1-NPN_), where [1-NPN] is the free concentration of 1-NPN, and K_1-NPN_ is the dissociation constant of the complex protein/1-NPN.

### Electroantennogram (EAG) and Olfactory Response Assays

The biologically attractive effects of chemicals with an ability to bind HparOBPs strongly were tested.

The EAG responses of virgin male/female antennae were measured after removing the tips of the three antennal lamellae (at approximately 1 mm) and separating them from each other. The chemicals used for the EAG and behavior assays were diluted in methanol (HPLC grade) to varying concentrations (0.1, 1, and 10 μg/μl), and methanol was used as a control. A 10 μl aliquot of each concentration was applied to a filter paper (25 × 8 mm). EAG responses were recorded for 5 s, with a stimulation interval of 30 s and a flow rate of 4 ml/s for both the stimulant and purge airflow. Each chemical was tested against six antennae, and each antenna was tested with three repeated stimulations. The EAG apparatus consisted of a signal acquisition system (IDAC-4), a micromanipulator assembly (INR-5), a stimulus controller (CS-05), and a system for outputting the EAG results (Syntech Company, Holland).

The behavioral responses of female and male adults to the putative ligands were tested using a Y-tube olfactometer in a dark room at 27 ± 1°C. A filter paper (25 × 8 mm) with 10 μl of the test compound was placed at the end of one arm of the Y-tube, with 10 μl of methanol at the end of the other arm (control tube). The airflow was 500 ml/min. Six replicates were performed for each stimulant with 10 healthy virgin adults in the main stem of the Y-tube, and 10 min was allowed for their distribution. The response rate was calculated according to the following equations: response rate = (T+C)/SUM and selective response rate = T/(T+C), where T represents the number of beetles in the treatment tube; C indicates the number of beetles in the control tube; and SUM is the number of beetles tested.

### Field Evaluation

According to the laboratory evaluation, in addition to the main sex pheromone component, L-leucine methyl ester, (*Z*)-3-hexenyl acetate was considered as a candidate compound for attracting DBC adults in the field. The tested chemicals were individually dissolved with methanol to 360 mg/ml. A dispenser was constructed using oil-free cotton wool with 360 mg of the tested chemical and stored in a freezer before use. Methanol was employed as a control. The treatments were as follows:

- L-leucine methyl ester alone, 360 mg/ml, 1 ml/dispenser
- (*Z*)-3-hexenyl acetate alone, 360 mg/ml, 1 ml/dispenser
- methanol alone as control, 99% purity, 1 ml/dispenser

All experiments were performed at the experimental station of the Shandong Peanut Research Institute, Lai Xi Wang Cheng, Qingdao, China. Traps were placed in middle of the field, and each trap was located 60 m from any other trap, so that the individual treatments were 60 m apart. To avoid cross-contamination, only one compound was tested at each sub-site at any time, and each compound was tested at only one sub-site (Isberg et al., 2017). Each trap was set to operate from 1 h before sunset until 1 h after sunset. Three traps (replicates) were selected for each treatment. The test period was June 1–20, 2017. As a rule, the traps were checked every day, and the individuals that were caught in all experiments were sexed.

## Results

### Characterization of Antenna OBP-Encoding Genes

A total of 30,338,129 paired-end reads were produced with a read length equal to 150 bp (Data Availability Statement: All the illumina sequencing data are available from the SRA database, accession number SRP148674). After low-quality filtering and adaptor cleaning, 30,297,575 filtered reads (representing 99.87% of total raw reads) were used for *de novo* assembly, resulting in a total of 106,562 contigs with an N50 length of 1,351 bp. The metrics of the DBC transcriptome assemblies were compared with those of the pine shoot beetle transcriptome (Zhu et al., 2012). The quality of these two transcriptome assemblies was comparable, indicating that the DBC assembly was suitable for downstream transcriptome analyses.

### Phylogenetic Analysis

**FIGURE 1:**
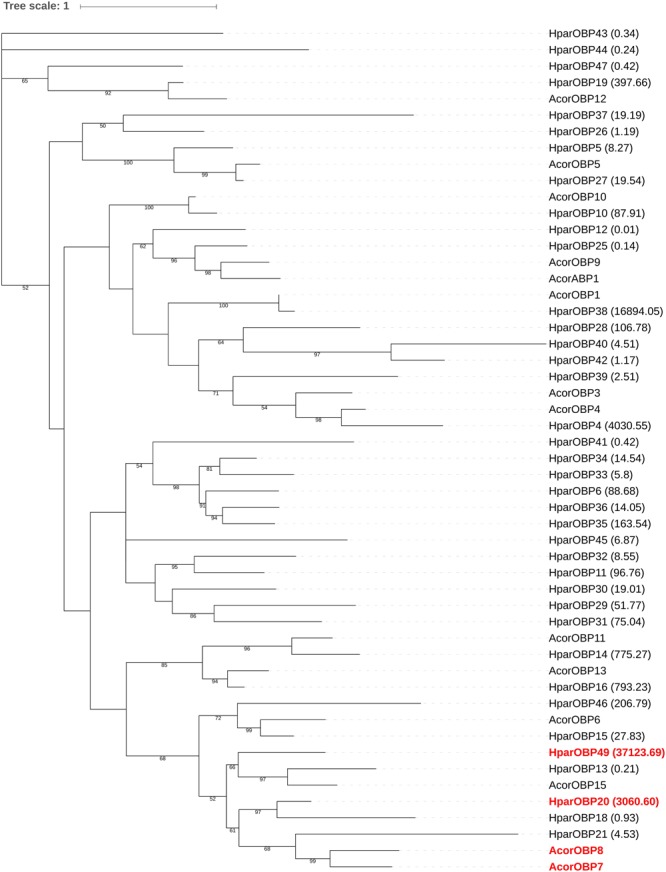
Phylogenetic tree of OBP genes in the DBC and *A. corpulenta*. The tree was constructed using IQ-TREE version 1.5.

A total of 48 HparOBP-encoding transcripts (containing 113–223 amino acids) were identified through BLAST searches. The OBPs of *Anomala corpulenta* and the DBC were employed to construct a phylogenetic tree (**Figure 1**). The phylogenetic tree showed that HparOBP20 (KR733566.1) and HparOBP49 (KR733548.1) were clustered with AcorOBP7 and AcorOBP8. Analysis of expression levels indicated that compared with other HparOBPs, HparOBP20 and HparOBP49 showed higher transcriptional activity. Therefore, HparOBP20 and HparOBP49 were selected for further analysis due to their tissue-specific expression pattern and high transcriptional activity in antennae (Ju et al., 2014).

### *In Vitro* Expression, Purification of Recombinant HparOBPs and Fluorescence Binding Assays of HparOBPs

**FIGURE 2:**
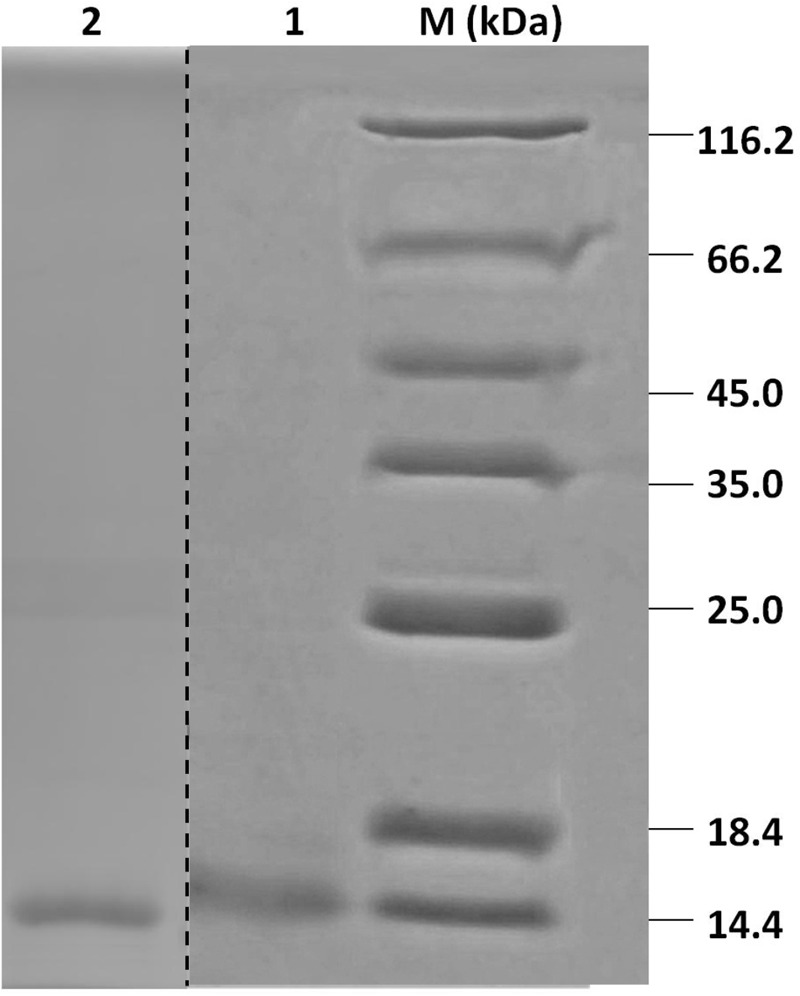
SDS-PAGE analyses of HparOBP purification. Protein markers (M) are labeled with sizes on the left and right sides. Lanes 1 and 2 show the purified proteins HparOBP20 and HparOBP49.

Recombinant HparOBPs were expressed in bacterial expression systems and purified using Ni ion affinity chromatography. SDS-PAGE analysis of the recombinant proteins showed that their molecular weights were 14–18 kDa, consistent with their predicted molecular masses (**Figure 2**).

**FIGURE 3:**
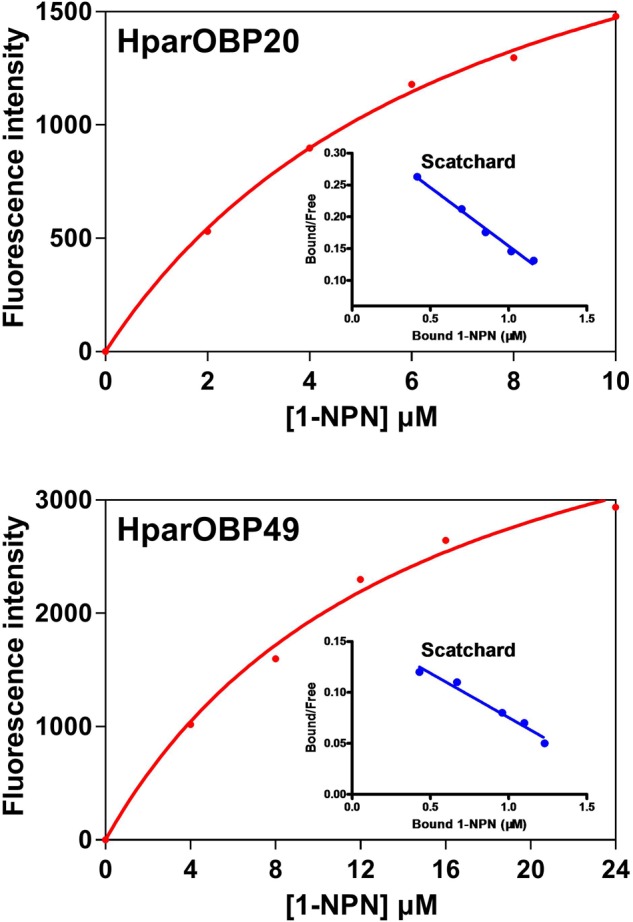
Saturation binding curves and relative Scatchard plots of the affinity of 1-NPN to HparOBPs. The dissociation constants of 1-NPN with the HparOBPs were 7.439 ± 1.45 (HparOBP20) and 14.67 ± 2.96 (HparOBP49), respectively.

NPN can be used as a probe in fluorescence binding assays of insect OBPs, and the binding properties of 1-NPN to OBPs have been well characterized (Sun et al., 2013; Zhuang et al., 2014; Li D.Z. et al., 2015). Therefore, 1-NPN was employed to establish saturation binding curves and Scatchard plots (**Figure 3**). The dissociation constants of 1-NPN with the HparOBPs, calculated using Scatchard plots, were 7.439 ± 1.45 (HparOBP20) and 14.67 ± 2.96 (HparOBP49), respectively.

**FIGURE 4:**
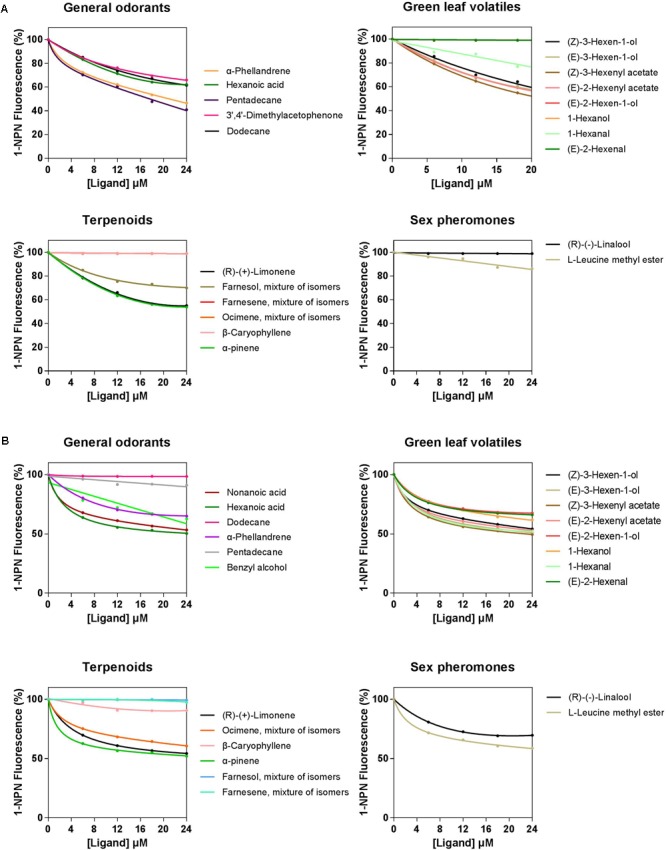
Competitive binding curves of host-associated volatiles and sex pheromone components with HparOBPs. **(A)** HparOBP20; **(B)** HparOBP49.

A total of 33 semiochemicals, including 31 host plant-associated volatiles and two sex pheromone components, were selected for fluorescence binding assays (**Figure 4** and **Table 1**). Among the 17 general odorants, HparOBP20 showed broad binding activity from Ki = 13.84 μM (pentadecane) to 40.60 μM (dodecane); HparOBP49 specifically bound to hexanoic acid with a Ki of 42.20 μM. Among the eight green-leaf volatiles (GLVs), (Z)-3-hexenyl acetate showed a high binding affinity to HparOBP20 and HparOBP49, with Ki values of 18.51 and 39.65 μM, respectively. In addition, (E)-2-hexenyl acetate, (Z)-3-hexen-1-ol and (E)-3-hexen-1-ol showed high binding affinities to HparOBP20, with Ki values of 23.25, 25.21, and 25.37 μM, respectively. Among the six terpenoids, HparOBP20 bound to α-pinene and (R)-(+)-limonene with Ki values of 22.41 and 23.99 μM. None of the tested terpenoids could displace 1-NPN bound to HparOBP49.

**Table 1:** Fluorescence-based competitive binding affinity of host-associated volatiles and sex pheromone components to recombinant HparOBPs.

Ligand	CAS Number	HparOBP20		HparOBP49	
		IC50 (μM)	Ki (μM)	IC50 (μM)	Ki (μM)
**General odorants**					
Dodecane (Cheng et al., 2010)	112-40-3	48.50	40.60	>50	–
Dodecyl aldehyde (Cheng et al., 2010)	112-54-9	>50	–	>50	–
Methyl benzoate (Chen et al., 2016)	93-58-3	>50	–	>50	–
Benzaldehyde (Leskey et al., 2014; Maeda et al., 2015)	100-52-7	>50	–	>50	–
Nonanoic acid (Shepherd and Sullivan, 2013)	112-05-0	>50	–	>50	–
α-Phellandrene (Siciliano et al., 2014)	99-83-2	21.09	17.65	>50	–
Nonanal (Fettig et al., 2012; Shepherd and Sullivan, 2013)	124-19-6	>50	–	>50	–
Benzyl alcohol (Shepherd and Sullivan, 2013)	100-51-6	>50	–	>50	–
Hexanoic acid (Green, 2014)	142-62-1	47.24	39.54	47.57	42.20
1-Octanol (Mukherjee et al., 2015)	111-87-5	30.18	25.26	>50	–
Methyl salicylate (Cheng et al., 2010; Maeda et al., 2015; Chen et al., 2016)	119-36-8	28.48	23.84	>50	–
1-Methylpyrrole (Burroni et al., 1997)	96-54-8	26.14	21.88	>50	–
Valeraldehyde (Germinara et al., 2016)	110-62-3	31.13	26.06	>50	–
4′-Ethylacetophenone (Zhang et al., 2013)	937-30-4	>50	–	>50	–
1,4-Cyclohexadiene (Li et al., 2009)	628-41-1	>50	–	>50	–
Pentadecane (Fan et al., 2015)	629-62-9	16.53	13.84	>50	–
3′,4′-Dimethylacetophenone (Pomonis et al., 1980)	3637-01-02	>50	–	>50	–
**Green-leaf volatiles**					
(Z)-3-Hexen-1-ol (Cheng et al., 2010; Maeda et al., 2015)	928-96-1	30.12	25.21	>50	–
(E)-3-Hexen-1-ol (Tang et al., 2012)	928-97-2	30.31	25.37	>50	–
(Z)-3-Hexenyl acetate (Cheng et al., 2010; Maeda et al., 2015)	3681-71-8	22.11	18.51	44.69	39.65
(E)-2-Hexenyl acetate (Allmann et al., 2013)	2497-18-9	27.78	23.25	>50	–
(E)-2-Hexen-1-ol (Fettig et al., 2012)	928-95-0	>50	–	>50	–
1-Hexanol (Shepherd and Sullivan, 2013)	111-27-3	>50	–	>50	–
1-Hexanal (Tang et al., 2012)	66-25-1	>50	–	>50	–
(E)-2-Hexenal (Fettig et al., 2012; Leskey et al., 2014; Chen et al., 2016)	6728-26-3	>50	–	>50	–
**Terpenoids**					
(R)-(+)-Limonene (Cheng et al., 2010; Leskey et al., 2014; Chen et al., 2016)	5989-27-5	28.66	23.99	>50	–
Farnesol, mixture of isomers (Cheng et al., 2010)	4602-84-0	>50	–	>50	–
Farnesene, mixture of isomers (Cheng et al., 2010; Delaney et al., 2013)	502-61-4	> 50	–	>50	–
Ocimene, mixture of isomers (Cheng et al., 2010)	13877-91-3	> 50	–	>50	–
β-Caryophyllene (Cheng et al., 2010; Chen et al., 2016)	87-44-5	>50	–	>50	–
α-Pinene (Cheng et al., 2010; Iqbal et al., 2014)	80-56-8	26.77	22.41	>50	–
**Sex pheromones**					
(R)-(-)-Linalool (Leal et al., 1993)	126-91-0	>50	–	>50	–
L-Leucine methyl ester (Leal et al., 1993)	7517-19-3	>50	–	>50	–

*Each value was obtained from three independent experiments. IC50 values labeled “>50” indicate that the binding affinity could not be calculated directly with the tested ligand concentrations. Therefore, the Ki values of these ligands are designated “-”.*

### EAG and Olfactory Responses to Host-Associated Volatiles and Sex Pheromone Components

**FIGURE 5:**
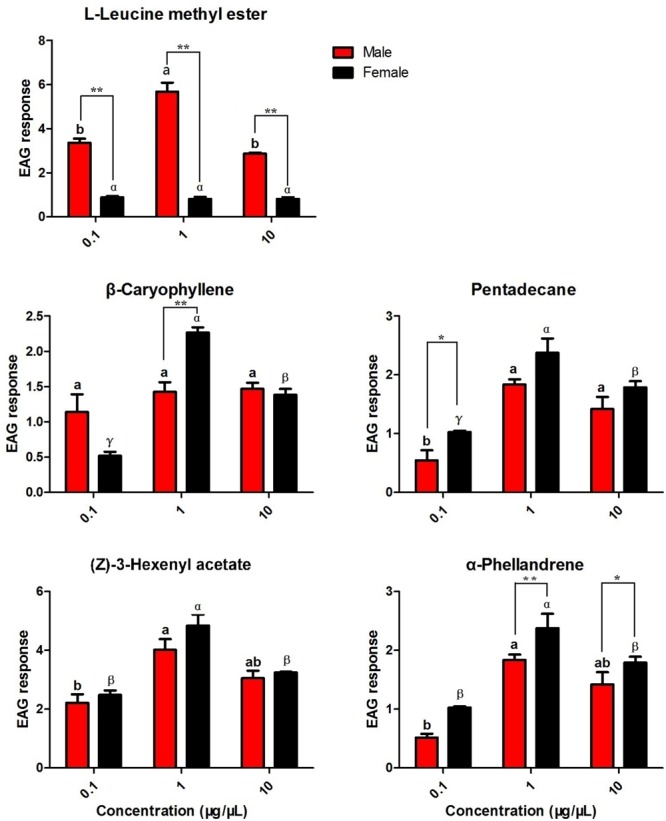
Electroantennogram (EAG) responses of male and female DBCs to host-associated volatiles and sex pheromone components. Mean ± SE (*N*= 6) after correction of the EAG with methanol. Significant differences between different chemicals were analysed through one-way analysis of variance (ANOVA) at a significance level of *P* < 0.05, and significant differences are indicated with different letters: a and b indicate males, and α, β, and γ indicated females. Asterisks indicate statistically significant differences between females and males (by Student’s *t-test*): ^∗^*P* < 0.05, ^∗∗^*P* < 0.01.

Based on the results of the fluorescence binding assays, five putative ligands of the recombinant HparOBPs were selected as candidates for EAG testing in both male and female antennae (**Figure 5**). In males, the highest responses were observed for L-leucine methyl ester and (*Z*)-3-hexenyl acetate at 1 μg/μl, with EAG responses of 5.68 and 4.02 mV, respectively. The highest response for females was observed for (*Z*)-3-hexenyl acetate at 1 μg/μl, with an EAG response of 4.84 mV. The dose-dependent EAG responses to (*Z*)-3-hexenyl acetate were similar in the two sexes. Significantly different EAG responses between the sexes were found for L-leucine methyl ester, with male antennae being more responsive than female antennae (*t* = 12.062, *P* < 0.01 for L-leucine methyl ester at 0.1 μg/μl; *t* = 11.635, *P* < 0.01 for L-leucine methyl ester at 1 μg/μl; and *t* = 19.231, *P* < 0.01 for L-leucine methyl ester at 10 μg/μl). At the concentration of 1 μg/μl, β-caryophyllene elicited a significantly higher response in female antennae than in male antennae (*t* = 5.350, *P* < 0.01).

**FIGURE 6:**
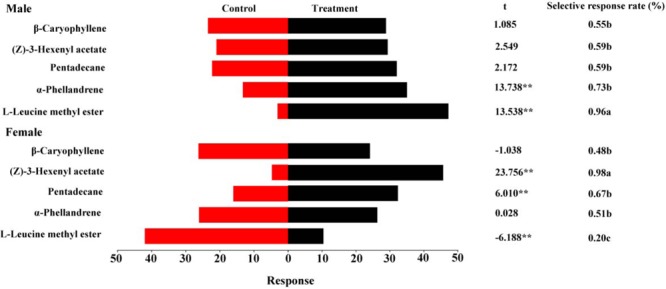
Behavioral responses of male and female DBCs to five putative HparOBP ligands at 1 μg/μl in a Y-tube olfactometer trial. A high response rate (greater than 80%) was observed for all DBC individuals. The indices were calculated using the following formulas: response = T/SUM or C/SUM, response rate = (T+C)/SUM and selective response rate = T/(T+C), where T represents the number of beetles in the treatment tube; C indicates the number of beetles in the control tube; and SUM is the number of beetles tested. Mean ± SE (*N*= 6). Asterisks indicate statistically significant differences between females and males (by Student’s *t-test*): ^∗^*P* < 0.05, ^∗∗^*P* < 0.01.

**Figure 6** summarizes the olfactory responses of DBC adults to the tested volatiles at 1 μg/μl. A good response rate (>80%) suggested that the tests were valid. Similar to the EAG responses, the highest selective response rate of females to (*Z*)-3-hexenyl acetate was 98%. A significantly higher selective response rate in males (96%) than in females was observed for L-leucine methyl ester. Significant differences in behavioral responses were observed between the controls and treatments for α-phellandrene and L-leucine methyl ester, with the treatment being more attractive than the control (*t* = 13.738, *P* < 0.01 for α-phellandrene; *t* = 13.538, *P* < 0.01 for L-leucine methyl ester). Females exhibited upwind movement into the volatiles containing pentadecane and (*Z*)-3-hexenyl acetate (*t* = 6.010, *P* < 0.01 for pentadecane, *t* = 23.756, *P* < 0.01 for (*Z*)-3-hexenyl acetate). L-Leucine methyl ester, an established sex pheromone component, attracted few female adults (*t* = -6.188, *P* < 0.01).

### Field Evaluation

**FIGURE 7:**
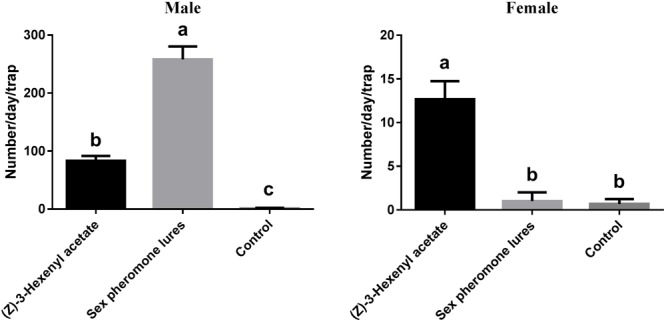
Total numbers of DBC captured in traps with different chemicals. Mean ± SE (*N*= 3). Significant differences between different treatments were analyzed via ANOVA at a significance level of *P* < 0.05, and significant differences are indicated with different letters.

Based on the EAG and olfactory responses, L-leucine methyl ester and (*Z*)-3-hexenyl acetate were selected for field evaluation (**Figure 7**). The results showed that all of the tested lures attracted more males than females. The sex pheromone resulted in significantly higher male catches than (*Z*)-3-hexenyl acetate (*F*_2,6_ = 272.1, *P* < 0.0001). For males, (*Z*)-3-hexenyl acetate yielded 83 ± 4.933 DBCs, and the sex pheromone yielded 258 ± 12.860. The average number of females per trap per day was 13 ± 1.202 using (*Z*)-3-hexenyl acetate (*F*_2,6_ = 74.18, *P* < 0.0001).

## Discussion

In this study, we focused on OBPs, which are relatively accessible targets for research, because they are small, soluble, stable and relatively easy to manipulate and modify (Brito et al., 2016; Leal, 2017; Zhu et al., 2017). *A. corpulenta* Motschulsky (Coleoptera: Scarabaeidae: Rutelinae) and DBC larvae, which are the main pests in many crop fields, exhibit overlapping active times, and adults of these species also overlap on some host plant species. Therefore, these pests may exhibit similar olfactory proteins in their olfactory systems, which could be the functional proteins interacting with plant volatiles. In *A. corpulenta*, AcroOBP7 and AcroOBP8 display antenna-specific expression (Li X. et al., 2015), and HparOBP20 and HparOBP49 exhibit antenna-specific expression in *H. parallela* (Ju et al., 2014). We have revised the nomenclature system of the *Holotrichia parallela* OBP genes in this paper. The OBP2 gene from the previous study (Ju et al., 2014) has been renamed HparOBP49. We hypothesize that these proteins are responsible for chemical communication, and the phylogenetic tree of *A. corpulenta* and *H. parallela* showed that HparOBP20 and HparOBP49 clustered with AcorOBP7 and AcorOBP8. Furthermore, an analysis of expression levels indicated that HparOBP20 and HparOBP49 showed higher transcriptional activity than that of other HparOBPs. Therefore, HparOBP20 and HparOBP49 were selected for further study. Their binding specificity may pave the way for the identification of active host plant volatiles.

To confirm the functions suggested by the phylogenetic tree, along with the tissue expression profiles and quantification analysis, the binding affinity of the two HparOBPs to 33 volatiles was determined using fluorescent binding assays. All the volatile compounds tested in this study were isolated from DBC host plants and may be biologically significant for the DBC. We found that HparOBP20 showed a broad spectrum of binding activity, and HparOBP49 specifically bound to general odorants and GLVs. Overall, HparOBP20 exhibited a high binding affinity to three volatiles (Ki < 20 μM): pentadecane, (*Z*)-3-Hexenyl acetate and α-phellandrene. However, all the volatiles tested in this study showed a relatively weak binding affinity (Ki > 20 μM) to HparOBP49. Compensation effects may exist between HparOBP20 and HparOBP49, as observed for *Cnaphalocrocis medinalis* OBP2 and OBP3 (Sun et al., 2016), and *Chrysopa pallens* OBP3, -6 and -10 (Li et al., 2017).

Among the three compounds that displayed a high binding affinity (Ki < 20 μM) to HparOBP20, (*Z*)-3-hexenyl acetate showed a higher affinity to both HparOBP20 and HparOBP49. (*Z*)-3-Hexenyl acetate is a GLV metabolized from one of the most abundant GLVs, (Z)-3-hexenal (Deng et al., 2004; Matsui, 2006; D’Auria et al., 2007; Allmann et al., 2013). (Z)-3-Hexenyl acetate is a common plant volatile released in large amounts after damage and plays important roles in insect-plant interactions (Arimura et al., 2008; Mumm and Dicke, 2010; Szendrei et al., 2011; von Arx et al., 2012). For example, a mixture of plant volatiles including (Z)-3-hexenyl acetate attracts the Colorado potato beetle, *Leptinotarsa decemlineata* Say (Visser, 1986), and the scarab beetle *Anomala octiescostata* Burmeister (Leal et al., 1994). Here, we tested the behavioral response and field attraction of the DBC to (Z)-3-hexenyl acetate, and a clear behavioral influence was observed in the EAG, Y-tube and field evaluations. The results were consistent with those of previous studies. Furthermore, (Z)-3-hexenyl acetate has been confirmed to activate olfactory sensory neurons (OSNs) expressing different sets of odorant receptor types on *Manduca sexta* female antennae (Allmann et al., 2013) and to enhance the responses of some insect species to sex pheromones (Deng et al., 2004; Varela et al., 2011; Ju et al., 2017). In the field, (Z)-3-hexenyl acetate mixed with sex pheromone in a 1:1 ratio increased the number of trap-caught females by 6- to 7-fold and the number of males by 20–30% compared with traps baited with sex pheromone alone (Reddy and Guerrero, 2000). Therefore, the synergistic effect between (Z)-3-hexenyl acetate and the sex pheromone requires further study. However, it is worth noting that, while (E)-2-hexenyl acetate displayed a lower binding affinity (Ki = 23.25) to HparOBP20, when it was employed in a trap along with the DBC sex pheromone, many DBCs were caught (Ju et al., 2017).

The general odorants pentadecane and α-phellandrene showed a higher binding affinity (Ki < 20 μM) to HparOBP20 and exerted a clear influence on behavior in the EAG and Y-tube assays but exhibited a low attractant ability in traps. Fluorescence binding assays often provide candidate compounds, but not all of the screened compounds exhibit biological activity in insects (Yi et al., 2018). AfunOBP1 from *Anopheles funestus* binds to 1-octen-3-ol, but when 1-octen-3-ol was used in a trap, only a few mosquito species were caught (Xu et al., 2010). However, pentadecane has been reported to bind to a *Locusta migratoria* OBP (Jiang et al., 2009), and the molecular docking results for α-phellandrene showed that it could tightly bind to the *Adelphocoris lineolatus* OBP6 pocket (Sun et al., 2017b). In the future, we may focus more research effort on these two odorants to obtain a greater number of DBC attractants.

## Author Contributions

QJ and XL analyzed and interpreted the data and performed the molecular examination. X-QG and LD performed the EAG and olfactory response examination. C-RS performed the field examination. M-JQ was a major contributor in writing the manuscript. All authors read and approved the final manuscript.

## Conflict of Interest Statement

The authors declare that the research was conducted in the absence of any commercial or financial relationships that could be construed as a potential conflict of interest.

## Footnotes

1 http://www.bioinformatics.babraham.ac.uk/projects/fastqc
