# Supplementary material for: Two Odorant-Binding Proteins of the Dark Black Chafer (Holotrichia parallela) Display Preferential Binding to Biologically Active Host Plant Volatiles
Source: Front Physiol. 2018 Jul 18;9:769. doi: 10.3389/fphys.2018.00769 (PMC6058716; doi:10.3389/fphys.2018.00769)
Supplement: Supplementary file 1 [file Table_1.DOCX]

Supplementary Material

Two Odorant-Binding Proteins of the Dark Black Chafer (*Holotrichia parallela*) Display Preferential Binding to Biologically Active Host Plant Volatiles

Qian Ju, Xiao Li, Xiao-qiang Guo, Long Du, Xiao-jing Jiang, Chen-ren Shi, Ming-jing Qu*

*** Correspondence:**Ming-jing Qu
E-mail: [13455277580@163.com](mailto:13455277580@163.com)

# Supplementary Data

**Table S1** Primer pairs used in the construction of HparOBP bacterial expression vectors

| Primer Name | Sequence (5’-3’) |
| --- | --- |
| HparOBP20-F1 | CGCGGATCCATGGATCAGGATTTCCTGAC |
| HparOBP20-R1 | CCGCTCGAGTTACATAGGCACCATTCCTG |
| HparOBP48-F1 | CGCGGATCCAGCATCGAGAAGTATATTG |
| HparOBP48-R1 | CCGCTCGAGTCATAACCCAATTAGTTTTTTG |

**Note:** All primers were synthesized by Shanghai Invitrogen Biotechnology Company (China).
